# A Case of Retroflexion of the Gravid Uterus with Over-Distension of the Bladder

**Published:** 1903-09-12

**Authors:** Robert Jardine

**Affiliations:** Professor of Midwifery in St. Mungo's College, Physician to the Glasgow Maternity Hospital


### Hospital Clinics and Medical Progress.

### A CASE OF RETROFLEXION OF THE GRAVID UTERUS WITH OVER-DISTENSION OF THE BLADDER.

A lecture delivered by Robert Jardine, M.D., F.R.S.E., Professor of Midwifery in St. Mungo's College, Physician to the Glasgow Maternity Hospital.

Gentlemen,—The history of the case I am about to show you in the labour-room is as follows: Yesterday I received a telephone message from a doctor in the country asking me if I would admit a case of his. On my asking the nature of the case he said that he and a friend had, after a consultation, come to the conclusion that it was an extra-uterine pregnancy. He said there was a tumour in the pelvis, and the patient's abdomen was much distended, although she was only four months pregnant. The patient was sent to hospital this morning.

Dr. Gairdner tells me that the patient's abdomen is nearly the size of a full-time pregnancy, and is occupied by a pyriform cystic tumour, the top of which extends above the umbilicus. The patient states that she first noticed the swelling about a week ago, and that it seemed to come in one night, during which time she had passed no urine. Since then she has at times been able to pass a little urine, but has not passed any since last night. There has not been any dribbling of urine. She states that she has been told that the child is outside the womb. She is a strong healthy-looking multipara.

I have no doubt as to what the condition is. It is certainly not an extra-uterine gestation. If the sac of an extra-uterine gestation suddenly increases in size in one night, the only thing that can have caused this is hæmorrhage; but this patient's pulse is quite strong, and she is not the least bit anæmic. The tumour in the abdomen I expect will be found to be the over-distended bladder, and the tumour in the pelvis the fundus of the retroflexed gravid uterus.

Here is the patient. You see she is a strong, healthy-looking woman, without any indications of anæmia. On inspecting the abdomen you see it is uniformly distended, just as in a pregnancy towards full time. On palpating the tumour it is found to be much the shape of a pregnant uterus, and the top of it is above the umbilicus. It is evidently cystic. There are no waves of contractions in it, and nothing like fœtal parts to be felt. On auscultating it no sounds are to be heard except the aortic pulsations. The patient suffers considerable discomfort, but there is no acute pain. On making a vaginal examination there is found a cystic globular swelling in the posterior fornix, and it is only with considerable difficulty that the os uteri can be reached, as it is very high and pushed well forward just above the symphysis pubis. I now pass a sterilised catheter, after the external parts have been thoroughly cleaned. You see there is a large quantity of perfectly healthy urine (126 ℥). I have drawn it all off, as I want to measure the exact amount; but, as you see, I put 2 pints of warm boracic solution into the bladder at once and leave it there. This is to prevent hæmorrhage from the mucous membrane of the bladder. If a bladder is suddenly emptied after it has been over-distended for some time, there may be violent hæmorrhage, and we wish to guard against this. Of course the same object would have been attained by only partly emptying the bladder, but I wished to find out how much urine there was.

You will now notice that the abdominal tumour has entirely disappeared, and the patient says she feels considerable relief. This is the first step in treating her case, and I might go on and deal with the uterus at once, but I shall leave it until to-morrow, as her doctor wishes to be present at any operation done. The bladder will be emptied entirely in about six hours, and the catheter passed regularly every six hours or so afterwards.

Now that the patient has been removed we can discuss her case freely. There is no necessity that she should be told of the mistaken diagnosis. When the uterus is once replaced, I shall tell her that everything has been put back into place, and that she will probably go to full time. It is unfortunate that she has been told that the child is outside of the womb, but she will probably think we have put it back again, and go away with a deep impression of our skill.

Now what has occurred here is that previous to the time of impregnation or subsequently to it the uterus has become displaced backwards. If there is any tendency to a posterior displacement before impregnation it is sure to be increased when the uterus grows in size. The uterus may have been in normal position when impregnated, and the displacement may have been caused by an accident. At first there is no particular discomfort, except that the patient may be conscious of a bearing-down feeling, but about the third month trouble with the bladder begins. As the uterus grows the cervix is pushed forwards until it presses against the neck of the bladder, and this pressure soon becomes so great that the patient is unable to empty her bladder, and she suffers from retention of urine. In most cases dribbling commences and the patient may imagine that she is passing plenty of urine. In the present case there was no dribbling, but the patient was able to pass a little urine from time to time, and thus prevent the bladder from bursting. The doctors were probably misled by the patient being able to pass urine. In the majority of cases, however, there is dribbling. In a case we had here a few weeks ago dribbling of urine had gone on for some weeks, and the contents of the bladder had become very septic. If the pregnancy goes on the uterus may right itself, *i.e.* grow up out of the pelvis, but so long as the bladder is full this is hardly likely to occur. In some cases, as in the one just referred to, a part of the uterus may rise out of the pelvis and a part remain incarcerated below the promontory. In other cases an abortion may occur, but from the position of the os (high up behind the symphysis), you can understand that it is very difficult for the uterus to empty itself. Occasionally the pressure upon the perineum is so great that sloughing occurs, and the uterine wall may become gangrenous.

The diagnosis of this condition is so easy that one wonders a mistake should ever be made. If the simple rule of always passing a catheter before examining the abdomen in the case of a tumour were carried out, this mistake would never be made.

*Treatment.*—The first part of the treatment we have already carried out in this case, viz., emptying the bladder with strict aseptic precautions, leaving enough fluid behind to obviate the risk of vesical hæmorrhage. To-morrow we shall replace the uterus. This may be done in several ways, by pressure from below. The fundus may be pushed up by the side of the promontory, by means of two fingers in the vagina, or by the forefinger in the vagina and the middle finger in the rectum. If the cervix is caught with forceps and pulled down at the same time the tilting upwards of the fundus will be assisted. If the patient is placed in the genu-pectoral position gravity will assist matters very much.

Occasionally it is impossible to raise the uterus. In such a case some advise bringing on an abortion by passing a sound into the uterus, which is not an easy operation to do, or by tapping the uterus through the posterior fornix. I am not in favour of either of these proceedings, and if I fail to replace in this case I shall open the abdomen and lift the uterus out of its bed and then close the abdomen. An abortion may occur after an abdominal section, but there is not a very great risk of this. After the uterus is replaced it may be necessary to support it with a ring pessary for about a month, but if the patient has reached the fourth month, as in this case, a support will probably not be needed.

